# Authors’ reply

**DOI:** 10.4103/0019-5413.73667

**Published:** 2011

**Authors:** Kantilal H Sancheti, Parag K Sancheti, Ashok K Shyam, Surendra Patil, Rajiv Joshi

**Affiliations:** *Sancheti Institute for Orthopaedics and Rehabilitation, Pune, India*

Sir,

We appreciate the thoughtful comments by the readers’^1^ in referencing our paper and the INDUS knee prosthesis.^2^

About readers’ inquiry regarding selection of patients with severe deformity, we can emphatically say that we have not excluded any joint that is fit for conventional total knee replacement. Only the knees that were grossly unstable and required a constrained implant were not implanted with INDUS knee prosthesis.

Preoperative factors like the preoperative range of motion, flexion deformity, body mass index, diagnosis etc are among the most important factors that define the postoperative outcome and these were the factors that lead to less than 100 degrees range in 24 patients in our study. The rationale of having a high flex design is not only achieving high flexion but to make achievement of this high flexion safe. For example, even with the conventional knee replacements many of our patients ignored the surgeon’s advice and continued sitting cross-legged which lead to early failure of these implants. The high flex features in INDUS knee makes this high flexion activity much safer than the conventional implant. The INDUS knee is able to achieve the mean range of motion comparable to the other high flexion knee joint which indicates the ability of the implant to achieve better range of motion within the given limitation of the pre-operative factor, although a detailed study with respect to these factors and comparison of the INDUS implant with the conventional design implants will be presented soon along with the midterm results.

We thoroughly agree that outcomes of fixed and mobile bearing are proven to be similar and so we designed INDUS to be a fixed bearing so as to make it more economical and suitable for the economic conditions of our country. Regarding stem extenders, the INDUS knee has an option of stem extenders in the tibial side but not on the femoral side. We personally do not think that requirement of stem extenders is a function of osteoporosis or inflammatory joint disease. We use stem extenders only in cases with bone defects that occupy more than 50% area of tibial condyle. We had none of the cases with such big defect in presented sample however we have performed more than forty cases with tibial stem extenders over the last two years.

We had no case of revision of INDUS knee so far in our early follow-up of two years study. Also tibial stem extenders are available for INDUS implant. Here we require making it very clear that we do not promote sitting cross-legged or squatting in our patients, but have merely reported their ability to do so at final follow up. We adequately warn our patients against this, however the High Flex features of the INDUS knee make noncompliant deep knee flexion in these patients much more safe thus preventing wear and providing longevity to the implant. In conclusion we say that, we have presented early results of INDUS knee prosthesis with mean range and associated complications in the present study which in our view is very satisfactory in terms of clinical, functional and financial benefit to our patients.

A complete detailed analysis of the pre-operative and intraoperative factors and their effect on outcome of INDUS implant is an ongoing study the results of which will be published soon.

## References

[1] Anand A, Raj RA (2010). The INDUS knee prosthesis: Prospective multicentric trial of posteriorly stabilized High-flex design: two years follow-up. Indian J Orthop.

[2] Sancheti KH, Laud NS, Bhende H, Reddy G, Pramod N, Mani JN (2009). The INDUS knee prosthesis: Prospective multicentric trial of a posteriorly stabilized high-flex design: 2 years follow-up. Indian J Orthop.

